# P093 Primary Sjögren syndrome complicated by type II cryoglobulinaemic vasculitis, immune-complex membranoproliferative glomerulonephritis, and bowel perforation in an elderly woman

**DOI:** 10.1093/rap/rkag113.123

**Published:** 2026-11-03

**Authors:** Lih Yin Chong, Anupama Nandagudi

**Affiliations:** Basildon University Hospital, Basildon, United Kingdom; Basildon University Hospital, Basildon, United Kingdom; Anglia Ruskin University, Cambridge, United Kingdom

## Abstract

**Introduction:**

Primary Sjögren syndrome (pSS) is a chronic autoimmune exocrinopathy characterised by sicca symptoms and systemic manifestations. Although most patients follow a benign course, a subset develop immune-complex–mediated complications including cryoglobulinaemic vasculitis and glomerulonephritis, conditions associated with significant morbidity and increased lymphoma risk. This case describes an 82-year-old woman with long-standing pSS who developed recurrent urticarial vasculitis, type II cryoglobulinemia, and biopsy-proven membranoproliferative glomerulonephritis (MPGN). Her disease course illustrates the diagnostic complexity of overlapping Sjögren–SLE features, the challenge of monitoring for lymphoproliferative transformation, and the importance of early recognition of renal involvement.

**Case description:**

An 82-year-old woman with a long-standing history of primary Sjögren syndrome, diagnosed in 2011 following presentation with sicca symptoms, photosensitivity, and a maculopapular rash, was referred for evaluation of progressive systemic involvement. Initial investigations demonstrated ANA positivity with strong anti-Ro60 and anti-Ro52 antibodies, and a minor salivary gland biopsy confirmed focal lymphocytic sialadenitis. She developed early systemic features including intermittent ankle swelling, steroid-responsive rashes, and persistently low complement levels. Cryoglobulinemia was identified within the first year of diagnosis, and she was managed with hydroxychloroquine, azathioprine, and tapering corticosteroids with good symptomatic control.

In 2015, she developed recurrent painful urticarial eruptions, and a skin biopsy confirmed urticarial vasculitis. Over the following years, her disease remained clinically stable, with intermittent sicca symptoms but no major organ involvement. Between 2016 and 2021, renal function remained preserved despite occasional microscopic haematuria and low-grade proteinuria.

Her condition deteriorated significantly in late 2022, when she presented with progressive bilateral lower-limb oedema, painful non-blanching purpura, and new-onset haematuria and proteinuria. Laboratory evaluation revealed markedly elevated rheumatoid factor (>240 U/mL), type II cryoglobulinemia with an IgM-κ paraprotein, and profoundly low C4 levels. Urine protein-creatinine ratio increased rapidly from 91 to 600 mg/mmol. A renal biopsy performed in early 2023 demonstrated immune-complex membranoproliferative glomerulonephritis consistent with cryoglobulinaemic vasculitis. She was treated with high-dose corticosteroids and rituximab, resulting in substantial improvement in renal parameters, cutaneous vasculitis, and functional status.

In mid-2024, she developed new gastrointestinal symptoms including abdominal pain, diarrhoea, weight loss, and progressive frailty. CT imaging revealed pneumoperitoneum, sigmoid thickening, and moderate ascites, raising concern for bowel perforation. The differential diagnosis included mesenteric ischaemia secondary to cryoglobulinaemic vasculitis, occult gastrointestinal malignancy, or lymphoma. Her clinical condition continued to decline despite supportive management.

**Discussion:**

• **Complex multisystem evolution of pSS:**

• This case demonstrates the long-term trajectory of primary Sjögren syndrome (pSS) when complicated by type II cryoglobulinemia.

• Although pSS is often mild, a subset develops chronic immune dysregulation with persistent hypocomplementemia, high rheumatoid factor, and cryoglobulin production.

• **Cryoglobulinemia as a high-risk phenotype**:

• Occurs in pSS patients and strongly predicts systemic vasculitis, renal involvement, and lymphoma.

• This patient exhibited high-risk markers early: low C4, strong anti-Ro positivity, and type II cryoglobulinemia with an IgM-κ monoclonal component.

• Persistent immune-complex formation and complement consumption over >10 years reflect ongoing B-cell activation.

• **Progression to cryoglobulinaemic vasculitis and renal disease:**

• Her 2022 deterioration with purpura, oedema, and rapidly rising proteinuria is typical of mixed cryoglobulinemia evolving into systemic vasculitis.

• Renal biopsy confirm immune-complex membranoproliferative glomerulonephritis (MPGM), the classical renal lesion in cryoglobulinaemic vasculitis.

• Profound C4 depletion and high rheumatoid factor supported active immune-complex disease.

• Rituximab produced significant improvement, highlighting the central role of B cell driven pathology.

• **Late gastrointestinal decline–vasculitis vs malignancy:**

• New abdominal pain, diarrhoea, weight loss, and pneumoperitoneum raised concern for mesenteric vasculitis with ischaemia or perforation.

• GI involvement in cryoglobulinaemic vasculitis is rare but recognised and may be catastrophic.

• Long-standing cryoglobulinemia, monoclonal IgM-κ, and recurrent vasculitis also raise suspicion for B-cell lymphoma, particularly marginal zone or lymphoplasmacytic types.

• Distinguishing vasculitic ischaemia from occult malignancy is challenging, especially in frail patients where invasive diagnostics are limited.

**Key learning points:**

1. Cryoglobulinemia in Sjögren syndrome signal systemic disease and high lymphoma risk. This patient had multiple predictors: low C4, positive RF, monoclonal IgM kappa, and recurrent vasculitis. Regular surveillance is essential.

2. Renal involvement in Sjögren syndrome requires early recognition. Rising urine PCR, haematuria, and low complement should prompt urgent nephrology referral. MPGN secondary to cryoglobulinemia responds best to B-cell–directed therapy such as rituximab.

3. Urticarial vasculitis with low complement suggests immune-complex disease rather than simple chronic urticaria. Skin biopsy is crucial. In this case, vasculitis changes were confirmed early, guiding immunosuppressive therapy.

4. GI symptoms in Sjögren syndrome should not be dismissed. Dysphagia, odynophagia, weight loss, and abdominal pain warrant endoscopic evaluation to exclude malignancy, lymphoma, or severe autoimmune involvement. Her late presentation with pneumoperitoneum and ascites underscores the risk of delayed investigation.

5. Overlap between Sjögren and SLE can complicate diagnosis. Low complement and immune-complex nephritis resemble SLE, but persistent anti-Ro/La positivity and negative dsDNA support Sjögren-dominant disease.

